# Deep learning for precise diagnosis and subtype triage of drug‐resistant tuberculosis on chest computed tomography

**DOI:** 10.1002/mco2.487

**Published:** 2024-03-10

**Authors:** Shufan Liang, Xiuyuan Xu, Zhe Yang, Qiuyu Du, Lingyu Zhou, Jun Shao, Jixiang Guo, Binwu Ying, Weimin Li, Chengdi Wang

**Affiliations:** ^1^ Department of Pulmonary and Critical Care Medicine State Key Laboratory of Respiratory Health and Multimorbidity, Targeted Tracer Research and Development Laboratory, Med‐X Center for Manufacturing, Frontiers Science Center for Disease‐related Molecular Network, West China Hospital, West China School of Medicine, Sichuan University Chengdu China; ^2^ Machine Intelligence Laboratory College of Computer Science Sichuan University Chengdu China; ^3^ Department of Laboratory Medicine West China Hospital, Sichuan University Chengdu China

**Keywords:** computed tomography, deep learning, drug‐resistant tuberculosis, multidrug‐resistant tuberculosis, rifampicin‐resistant tuberculosis

## Abstract

Deep learning, transforming input data into target prediction through intricate network structures, has inspired novel exploration in automated diagnosis based on medical images. The distinct morphological characteristics of chest abnormalities between drug‐resistant tuberculosis (DR‐TB) and drug‐sensitive tuberculosis (DS‐TB) on chest computed tomography (CT) are of potential value in differential diagnosis, which is challenging in the clinic. Hence, based on 1176 chest CT volumes from the equal number of patients with tuberculosis (TB), we presented a Deep learning‐based system for TB drug resistance identification and subtype classification (DeepTB), which could automatically diagnose DR‐TB and classify crucial subtypes, including rifampicin‐resistant tuberculosis, multidrug‐resistant tuberculosis, and extensively drug‐resistant tuberculosis. Moreover, chest lesions were manually annotated to endow the model with robust power to assist radiologists in image interpretation and the Circos revealed the relationship between chest abnormalities and specific types of DR‐TB. Finally, DeepTB achieved an area under the curve (AUC) up to 0.930 for thoracic abnormality detection and 0.943 for DR‐TB diagnosis. Notably, the system demonstrated instructive value in DR‐TB subtype classification with AUCs ranging from 0.880 to 0.928. Meanwhile, class activation maps were generated to express a human‐understandable visual concept. Together, showing a prominent performance, DeepTB would be impactful in clinical decision‐making for DR‐TB.

## Graphical abstract

We proposed a Deep learning‐based system for tuberculosis (TB) drug resistance identification and subtype classification, DeepTB, achieving prominent performance in chest abnormality recognition, drug‐resistant tuberculosis (DR‐TB) diagnosis, and subtype triage on computed tomography (CT) volumes. DeepTB holds great promise to help physicians with TB management and a further purpose to improve general welfare.

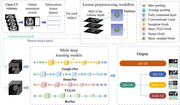

## 1 INTRODUCTION

Tuberculosis (TB), induced by the bacillus *Mycobacterium tuberculosis*, is one of the leading causes of infectious mortality and results in approximately 10.6 million cases and 1.3 million deaths in 2022.^1^ Drug‐resistant tuberculosis (DR‐TB) serves as a predominant factor hindering the World Health Organization (WHO) End TB strategy realization on account of the more complex regimens with higher risk of adverse effects and worse outcomes.^2^ With 410,000 individuals developed multidrug‐resistant or rifampicin‐resistant tuberculosis (MDR/RR‐TB) and 57% of cases untreated,^1^ DR‐TB management is far from the global target. Moreover, approximately 25% of antimicrobial resistance‐related deaths are due to rifampicin‐resistant tuberculosis (RR‐TB).^3^ Extensively drug‐resistant tuberculosis (XDR‐TB), the most advanced subtype of DR‐TB,^4^ with less than half successful treatment and being more prone to long‐term sequelae, is of great concern as well.^5^, ^6^ Consequently, DR‐TB remains a substantial threat to public hygiene, and prompt diagnosis and optimal therapeutic strategy‐making based on the susceptibility profiles of DR‐TB are crucial for TB supervision.

Thus far, culture‐based phenotypic drug‐susceptibility testing (DST) is indicated as the primary reference standard of drug resistance analysis but is technically difficult, requiring strict operation and quality management. More to the point, this procedure is restricted by the slow reproduction of *Mycobacterium tuberculosis*, resulting in appropriate intervention delays due to the drawbacks of being time consuming for weeks.^7^, ^8^ Genetic approaches, such as line‐probe assays and whole genome sequencing, which decide phenotypic resistance via the genotypes of strains, although reducing turnaround time, are bacterial load‐ and facility‐dependent.^8^ Furthermore, the association between variations and novel or rare anti‐TB agents remains to be analyzed, potentially leading to diagnostic mishaps.^9^

Besides laboratory tests, radiology plays a pivotal role in TB screening and diagnosis owing to its accessibility and efficiency.^10^ Previous evidence has revealed that certain manifestations on chest computed tomography (CT) volumes, such as pulmonary nodules, thick‐wall cavities, consolidations, bronchiectasis, whole‐lung involvement, disseminated lesions, and so on, are more frequently observed in DR‐TB.^11^, ^12^, ^13^ Additionally, cavities and lesion extent are characterized differently among patients with RR‐TB, multidrug‐resistant tuberculosis (MDR‐TB), and XDR‐TB, implying the diagnostic potential of chest CT imaging.^14^

Currently, image‐based artificial intelligence (AI) systems have been proposed for the detection and activity assessment of TB, achieving, or even surpassing the performance of human physicians.^15^, ^16^, ^17^, ^18^, ^19^, ^20^ For automated diagnosis of DR‐TB, chest X‐ray (CXR), a two‐dimensional projection consisting of overlapped anatomical structures, was commonly utilized, whereas CT imaging, a three‐dimensional (3D) reconstruction presenting higher diagnostic reliability, has been seldom reported.^21^ Moreover, moderate performance and small‐scale datasets hinder the practicality and generality of those models.^22^, ^23^

Therefore, in this study, we proposed a superior semi‐supervised multitask deep learning model, named Deep learning‐based system for TB drug resistance identification and subtype classification (DeepTB), based on chest CT volumes and large‐scale dataset‐pretrained convolutional neural network (CNN)‐ResNet, for simultaneous pulmonary abnormality recognition, diagnosis, and subtype classification of DR‐TB accurately, with a further purpose to support optimal clinical decision‐making.

## 2 RESULTS

### 2.1 Demographic characteristics of included patients and dataset composition

**FIGURE 1:**
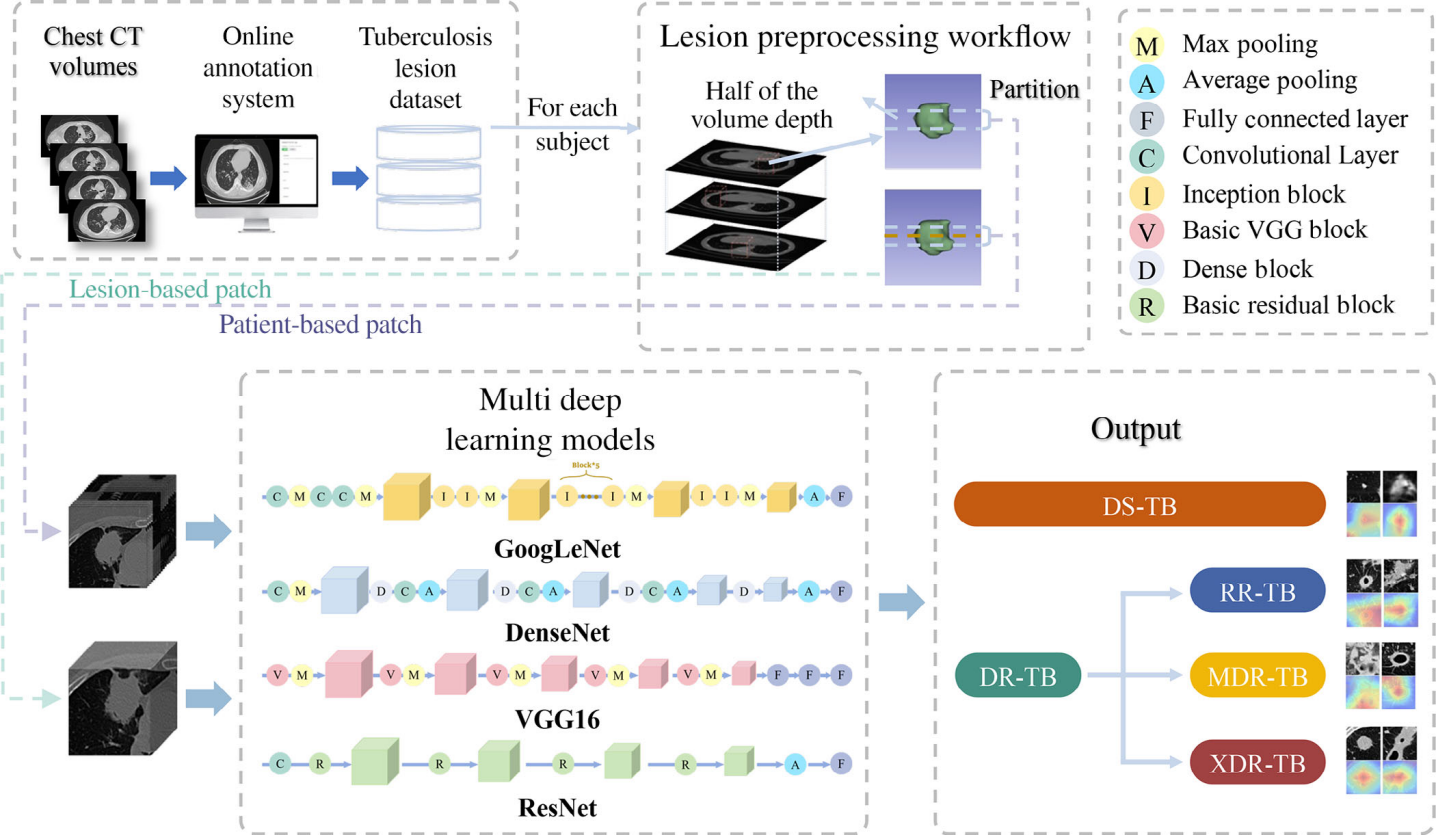
Flow chart of the deep learning model construction. CT, computed tomography; DR‐TB, drug‐resistant tuberculosis; DS‐TB, drug‐sensitive tuberculosis; MDR‐TB, multidrug‐resistant tuberculosis; RR‐TB, rifampicin‐resistant tuberculosis; XDR‐TB, extensively drug‐resistant tuberculosis.

The overall workflow was provided in Figure 1.^24^ And characteristics of the included patients and the selection process were respectively summarized in Table 1 and Figure S1. Following the inclusion criteria, 1751 patients were initially enrolled. After the exclusion process, 1176 patients were confirmed selected, consisting of 390 (33.2%) individuals with DR‐TB and 786 (66.8%) patients with drug‐sensitive tuberculosis (DS‐TB). There were differences between the two cohorts. The DR‐TB cohort had a mean age of 43.3 years and a male proportion of 66.2%. Nevertheless, the DS‐TB group held a higher average age of 45.3 years and a declined percentage of males of 57%, consistent with previous studies, indicating the risk factors associated with DR‐TB of being younger and male sex.^25^, ^26^

**TABLE 1:** Summary of the included patients and labeled chest abnormalities.

Characteristic	DR‐TB	DS‐TB	Total
No. of patients	390 (33.2)	786 (66.8)	1176
Age (years)^a^	43.3 ± 16.6	45.3 ± 18	44.9 ± 17.8
Sex (male)	258 (66.2)	448 (57)	706 (60)
Three major subtypes of DR‐TB	252	—	252
RR‐TB	117 (46.4)	—	117 (46.4)
MDR‐TB	124 (49.2)	—	124 (49.2)
XDR‐TB	11 (4.4)	—	11 (4.4)
Chest abnormality	432	1231	1663
Nodule	80 (18.5)	**473 (38.4)**	553 (33.3)
Patchy shadow	**109 (25.2)**	248 (20.1)	357 (21.5)
Cavity	**93 (21.5)**	177 (14.4)	270 (16.2)
Consolidation	46 (10.6)	**196 (15.9)**	242 (14.5)
Pleural effusion	**40 (9.3)**	30 (2.4)	70 (4.2)
Bronchiectasis	**37 (8.6)**	29 (2.4)	66 (4)
Miliary nodule	6 (1.4)	21 (1.7)	27 (1.6)
GGO	3 (0.7)	17 (1.4)	20 (1.2)
Mass	3 (0.7)	12 (1)	15 (0.9)
Others	15 (3.5)	28 (2.3)	43 (2.6)

Data in parentheses are percentages. Higher numbers and percentages of labeled chest abnormalities when there were obvious differences between the two groups are bolded.

Abbreviations: DR‐TB, drug‐resistant tuberculosis; DS‐TB, drug‐sensitive tuberculosis; GGO, ground‐glass opacity; MDR‐TB, multidrug‐resistant tuberculosis; RR‐TB, rifampicin‐resistant tuberculosis; XDR‐TB, extensively drug‐resistant tuberculosis.

^a^ Data are presented as means ± standard deviations.

In the subtask of DR‐TB subtype classification, three major types, including RR‐TB, MDR‐TB, XDR‐TB, were analyzed. As for the grouping principle, we followed the drug resistance definition recommended by WHO.^27^ The respective number of patients with RR‐TB, MDR‐TB, and XDR‐TB in this task was 117 (46.4%), 124 (49.2%), and 11 (4.4%).

In terms of DR‐TB diagnosis, the CT volumes were randomly split in the ratio of 7:2:1 into training (*n* = 823), validation (*n* = 235), and test (*n* = 118) sets, without overlapping among them. The dataset partitioning strategy in DR‐TB subtyping followed the same ratio.

### 2.2 Characteristics of chest abnormalities

For pulmonary abnormality detection, we manually marked 1663 lesion labels, which were then divided at the ratio of 7:2:1 for training (*N* = 1164), validation (*N* = 333), and test (*N* = 166). In the annotation step, we framed predominant chest abnormalities on the CT scans with rectangles along with lesion labels, including nodule, patchy shadow, cavity, consolidation, pleural effusion, bronchiectasis, miliary nodule, mass, ground‐glass opacity (GGO), and others. It needs to be emphasized that not all the lesions were marked. Instead, owing to the complexity of TB imaging and the resultant laborious workload, we mainly concentrated on critical abnormalities that meaningfully contributed to the final diagnosis and utilized a semi‐supervised learning method to autonomously generate pseudo‐labels for the remaining unlabeled data (detailed model development was introduced in the *Materials and Methods* section).

**FIGURE 2:**
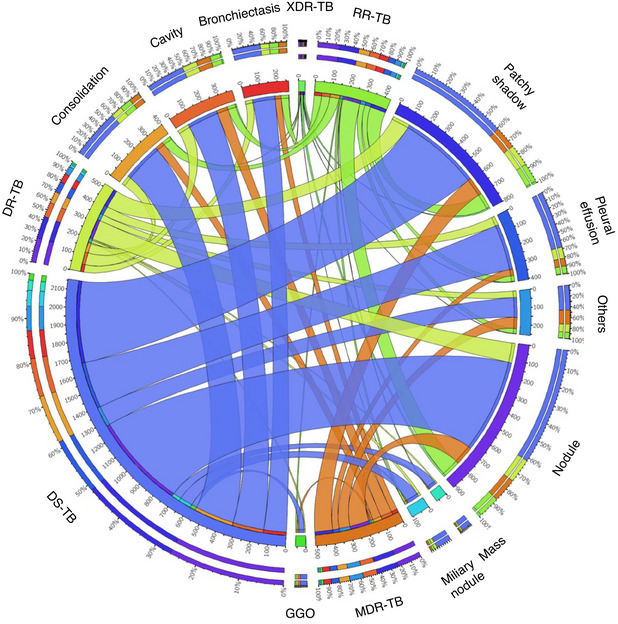
Circos of the correlation between chest abnormalities and types of tuberculosis drug resistance. Outer circle demonstrates the total proportion of the correlation and inner circle presents the relationship. The wider strip corresponds to stronger correlation. DR‐TB, drug‐resistant tuberculosis; DS‐TB, drug‐sensitive tuberculosis; GGO, ground‐glass opacity; MDR‐TB, multidrug‐resistant tuberculosis; RR‐TB, rifampicin‐resistant tuberculosis; XDR‐TB, extensively drug‐resistant tuberculosis.

After the manual labeling and statistics, patchy shadows (25.2%) and cavities (21.5%) were most dominant on CT scans in patients with DR‐TB, meanwhile holding a higher incidence of pleural effusion (9.3%) and bronchiectasis (8.6%) than DS‐TB. Nevertheless, in the DS‐TB cohort, nodules (38.4%) and consolidations (15.9%) were more frequently observed (Table 1). Additionally, the Circos which presented the correlation between types of drug resistance and chest pathologies was depicted in Figure 2.

### 2.3 Model performance in chest abnormality detection

**FIGURE 3:**
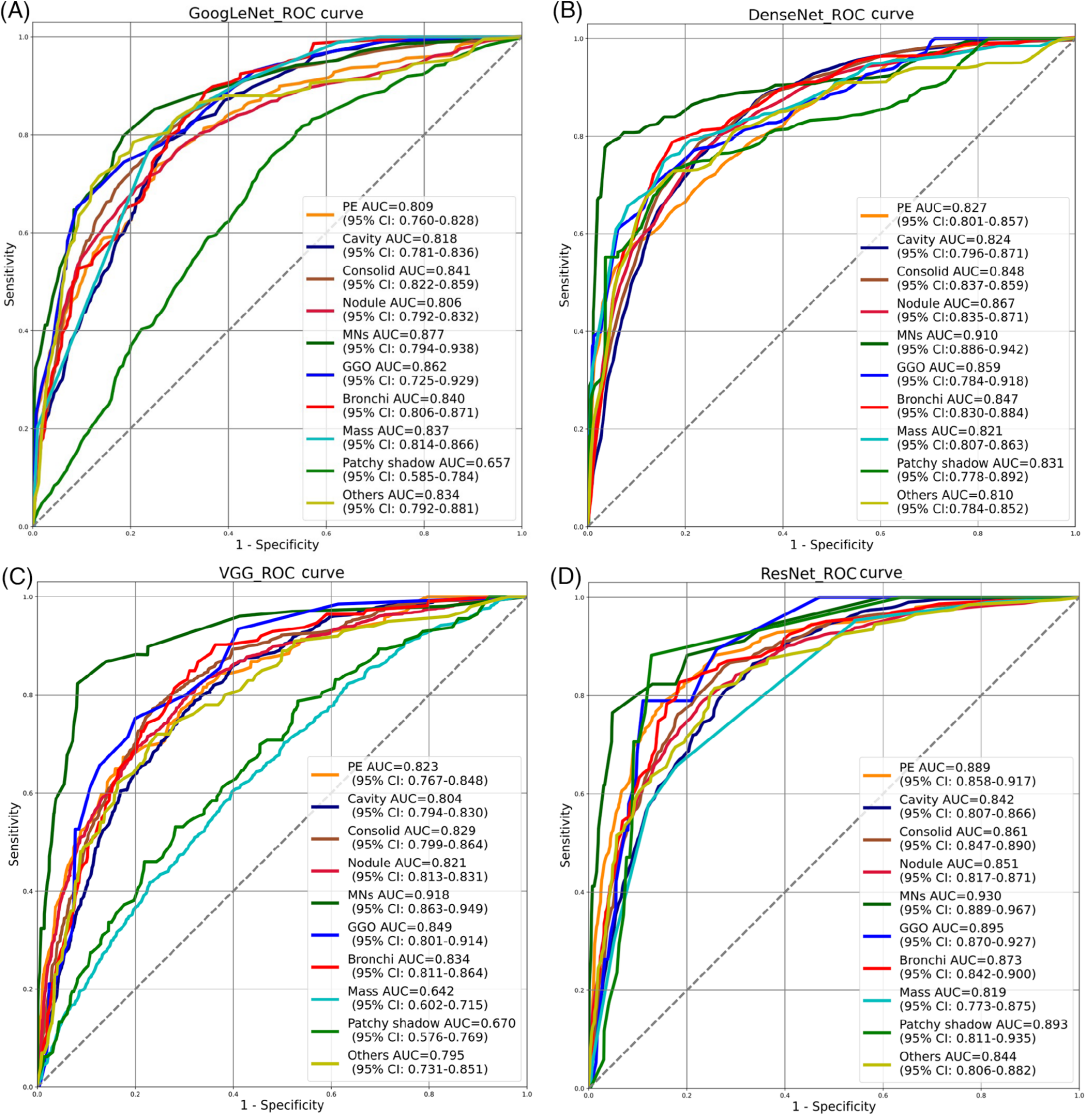
Receiver operating characteristic (ROC) curves of chest abnormality detection of GoogleNet (A), DenseNet (B), VGG16 (C), and ResNet (D) in the test set. AUC, area under the curve; Bronchi, bronchiectasis; CI, confidence interval; Consolid, consolidation; GGO, ground‐glass opacity; MNs, miliary nodules; PE, pleural effusion.

For the model development, in the first step, we investigated whether the model could assist radiologists in multifocal chest abnormality interpretation via automated localization and qualitative determination. Receiver operating characteristic (ROC) curves of the four models, including GoogLeNet, DenseNet, VGG16, and ResNet, in the task were shown in Figure 3. For the different thoracic abnormalities, GoogLeNet, DenseNet, VGG16, and ResNet achieved respective area under the curve (AUC) up to 0.877 (95% confidence interval [CI]: 0.794, 0.938), 0.910 (95% CI: 0.886, 0.942), 0.918 (95% CI: 0.863, 0.949), and 0.930 (95% CI: 0.889, 0.967).

ResNet performed best in this task overall, with the highest detection AUCs of 0.893 (95% CI: 0.811, 0.935) for patchy shadow, 0.842 (95% CI: 0.807, 0.866) for cavity, 0.861 (95% CI: 0.847, 0.890) for consolidation, 0.889 (95% CI: 0.858, 0.917) for pleural effusion, 0.873 (95% CI: 0.842, 0.900) for bronchiectasis, 0.930 (95% CI: 0.889, 0.967) for miliary nodule, and 0.895 (95% CI: 0.870, 0.927) for GGO. DenseNet accidentally exceeded ResNet in nodule recognition with an AUC of 0.867 (95% CI: 0.835, 0.871) while ResNet showed a slightly lower AUC of 0.851 (95% CI: 0.817, 0.871). Moreover, GoogLeNet presented the highest AUC of 0.837 (95% CI: 0.814, 0.866) for mass detection (Table 2). One intriguing phenomenon was that almost all models presented the most accurate performance in miliary nodule identification. A likely candidate was the distinctive feature of miliary nodule being uniform in distribution, size, and density, along with the widespread lung field involvement.^28^

**TABLE 2:** Performance of four models in chest abnormality detection of tuberculosis in the test set.

Chest abnormality	Model	Accuracy (95% CI)	Recall (95% CI)	Specificity (95% CI)	Precision (95% CI)	F1‐score (95% CI)	AUC (95% CI)
Nodule	GoogLeNet	0.648 (0.630, 0.666)	0.880 (0.860, 0.899)	0.854 (0.837, 0.872)	0.543 (0.519, 0.566)	0.671 (0.653, 0.689)	0.806 (0.792, 0.832)
	DenseNet	0.730 (0.713, 0.747)	**0.857** (0.836, 0.878)	**0.867** (0.850, 0.884)	0.624 (0.599, 0.648)	0.722 (0.705, 0.739)	**0.867** (0.835, 0.871)
	VGG16	0.724 (0.707, 0.741)	0.838 (0.815, 0.860)	0.852 (0.834, 0.869)	0.620 (0.595, 0.645)	0.712 (0.695, 0.730)	0.821 (0.813, 0.831)
	ResNet	0.862 (0.741, 0.878)	0.854 (0.832, 0.885)	0.881 (0.853, 0.886)	0.648 (0.620, 0.701)	0.738 (0.714, 0.761)	0.851 (0.817, 0.871)
Patchy shadow	GoogLeNet	0.939 (0.930, 0.948)	0.259 (0.147, 0.371)	0.874 (0.810, 0.907)	0.207 (0.193, 0.284)	0.230 (0.116, 0.507)	0.657 (0.585, 0.784)
	DenseNet	0.949 (0.940, 0.957)	0.458 (0.318, 0.597)	0.916 (0.904, 0.964)	0.406 (0.322, 0.563)	0.430 (0.214,0.671)	0.831 (0.778, 0.892)
	VGG16	0.876 (0.864, 0.889)	0.359 (0.247, 0.471)	0.893 (0.860, 0.926)	0.303 (0.297, 0.341)	0.329 (0.274, 0.415)	0.670 (0.576, 0.769)
	ResNet	**0.895** (0.881, 0.901)	0.762 (0.560, 0.963)	**0.882** (0.871, 0.890)	0.671 (0.424, 0.732)	0.541 (0.481, 0.614)	**0.893** (0.811, 0.935)
Cavity	GoogLeNet	0.847 (0.833, 0.860)	0.186 (0.150, 0.222)	0.855 (0.840, 0.869)	0.680 (0.598, 0.763)	0.292 (0.275, 0.310)	0.818 (0.781, 0.836)
	DenseNet	0.826 (0.811, 0.840)	0.623 (0.565, 0.655)	0.918 (0.906, 0.930)	0.491 (0.450, 0.532)	0.549 (0.524, 0.563)	0.824 (0.796, 0.871)
	VGG16	0.811 (0.796, 0.826)	0.498 (0.451, 0.544)	0.895 (0.882,0.907)	0.450 (0.406, 0.494)	0.473 (0.454, 0.492)	0.804 (0.794, 0.830)
	ResNet	0.809 (0.794, 0.827)	0.627 (0.581, 0.674)	**0.916** (0.901, 0.924)	0.462 (0.422, 0.513)	0.560 (0.521, 0.593)	**0.842** (0.807, 0.866)
Consolidation	GoogLeNet	0.827 (0.813, 0.842)	0.633 (0.589, 0.677)	0.918 (0.907, 0.930)	0.502 (0.461, 0.543)	0.560 (0.541, 0.579)	0.841 (0.822, 0.859)
	DenseNet	0.823 (0.808, 0.838)	0.697 (0.654, 0.739)	0.930 (0.919, 0.941)	0.493 (0.454, 0.532)	0.577 (0.558, 0.596)	0.848 (0.837, 0.859)
	VGG16	0.776 (0.760, 0.792)	0.736 (0.696, 0.777)	0.934 (0.924, 0.944)	0.418 (0.384, 0.452)	0.533 (0.514, 0.552)	0.829 (0.799, 0.864)
	ResNet	0.794 (0.778, 0.811)	0.774 (0.731, 0.804)	0.847 (0.836, 0.854)	0.458 (0.411, 0.494)	0.574 (0.571, 0.631)	**0.861** (0.847, 0.890)
Pleural effusion	GoogLeNet	0.872 (0.859, 0.885)	0.506 (0.431, 0.580)	0.962 (0.955, 0.970)	0.261 (0.214, 0.308)	0.344 (0.326, 0.363)	0.809 (0.760, 0.828)
	DenseNet	0.879 (0.866, 0.891)	0.580 (0.507, 0.654)	0.968 (0.961, 0.975)	0.292 (0.244, 0.340)	0.388 (0.370, 0.407)	0.827 (0.801, 0.857)
	VGG16	0.878 (0.865, 0.890)	0.529 (0.455, 0.603)	0.964 (0.957, 0.972)	0.279 (0.230, 0.327)	0.365 (0.347, 0.384)	0.823 (0.767, 0.848)
	ResNet	**0.890** (0.882, 0.906)	0.678 (0.614, 0.758)	**0.875** (0.861, 0.891)	0.372 (0.341, 0.454)	0.483 (0.441, 0.554)	**0.889** (0.858,0.917)
Bronchiectasis	GoogLeNet	0.895 (0.883, 0.906)	0.504 (0.412, 0.597)	0.826 (0.805, 0.845)	0.206 (0.158, 0.253)	0.292 (0.275, 0.310)	0.840 (0.806, 0.871)
	DenseNet	0.825 (0.811, 0.840)	0.796 (0.692, 0.848)	0.852 (0.832, 0.899)	0.170 (0.135, 0.200)	0.280 (0.258, 0.292)	0.847 (0.830, 0.884)
	VGG16	0.817 (0.802, 0.832)	0.655 (0.567, 0.743)	0.801 (0.735, 0.927)	0.144 (0.114, 0.174)	0.236 (0.220, 0.252)	0.834 (0.811, 0.864)
	ResNet	0.847 (0.834, 0.872)	0.764 (0.697, 0.851)	**0.890** (0.872, 0.904)	0.202 (0.168, 0.262)	0.312 (0.261, 0.344)	**0.873** (0.842, 0.900)
Miliary nodule	GoogLeNet	0.950 (0.942, 0.958)	0.500 (0.332, 0.668)	0.993 (0.990, 0.996)	0.130 (0.072, 0.187)	0.206 (0.191, 0.222)	0.877 (0.794, 0.938)
	DenseNet	0.953 (0.945, 0.961)	0.588 (0.423, 0.754)	0.894 (0.876, 0.926)	0.155 (0.092, 0.217)	0.245 (0.227, 0.260)	0.910 (0.886, 0.942)
	VGG16	0.926 (0.916, 0.936)	0.706 (0.553, 0.859)	0.896 (0.843, 0.928)	0.115 (0.072, 0.158)	0.198 (0.182, 0.213)	0.918 (0.863, 0.949)
	ResNet	**0.917** (0.901, 0.935)	**0.894** (0.858, 1.000)	**0.937** (0.905, 0.967)	0.384 (0.153, 0.545)	0.601 (0.538, 0.697)	**0.930** (0.889, 0.967)
Mass	GoogLeNet	**0.968** (0.961, 0.975)	0.500 (0.098, 0.901)	**0.858** (0.837, 1.000)	0.436 (0.392, 0.476)	0.466 (0.421, 0.505)	**0.837** (0.814, 0.866)
	DenseNet	0.966 (0.951, 0.981)	0.514 (0.197, 0.910)	0.879 (0.836, 0.914)	0.353 (0.263, 0.444)	0.419 (0.273, 0.524)	0.821 (0.807, 0.863)
	VGG16	0.944 (0.935, 0.953)	0.633 (0.552, 0.711)	0.928 (0.907, 0.959)	0.381 (0.271, 0.491)	0.476 (0.275, 0.684)	0.642 (0.602, 0.715)
	ResNet	0.924 (0.908, 0.947)	0.788 (0.711, 0.942)	0.904 (0.884, 0.937)	0.172 (0.042, 0.583)	0.371 (0.141, 0.698)	0.818 (0.773, 0.875)
GGO	GoogLeNet	0.884 (0.872, 0.897)	0.684 (0.475, 0.893)	0.897 (0.895, 0.899)	0.241 (0.208, 0.308)	0.356 (0.297, 0.385)	0.862 (0.725, 0.938)
	DenseNet	0.949 (0.941, 0.958)	0.474 (0.199, 0.643)	0.916 (0.881, 0.936)	0.270 (0.213, 0.326)	0.344 (0.327, 0.436)	0.859 (0.784, 0.918)
	VGG16	0.843 (0.829, 0.857)	0.684 (0.475, 0.893)	0.847 (0.795, 0.929)	0.431 (0.294, 0.577)	0.529 (0.378, 0.673)	0.849 (0.801, 0.914)
	ResNet	**0.961** (0.901, 0.981)	**0.987** (0.894, 1.000)	**0.911** (0.726, 0.995)	0.212 (0.114, 0.517)	0.391 (0.104, 0.687)	**0.895** (0.870, 0.927)
Others	GoogLeNet	0.841 (0.827, 0.855)	0.730 (0.643, 0.817)	0.877 (0.843, 0.901)	0.158 (0.124, 0.191)	0.259 (0.243, 0.276)	0.834 (0.792, 0.881)
	DenseNet	0.853 (0.840, 0.867)	0.690 (0.600, 0.781)	0.765 (0.713, 0.791)	0.164 (0.129, 0.199)	0.264 (0.247, 0.281)	0.810 (0.784, 0.852)
	VGG16	0.867 (0.854, 0.880)	0.520 (0.422, 0.618)	0.848 (0.813, 0.894)	0.147 (0.110, 0.184)	0.230 (0.213, 0.246)	0.795 (0.731, 0.851)
	ResNet	**0.887** (0.851, 0.912)	0.643 (0.551, 0.745)	**0.884** (0.871, 0.894)	0.194 (0.135, 0.229)	0.342 (0.218, 0.389)	**0.844** (0.806, 0.882)

The highest AUC value, along with corresponding accuracy, recall, and specificity when they were over 0.85 of each chest abnormality were bolded.

Abbreviations: AUC, area under the curve; CI, confidence interval; GGO, ground‐glass opacity.

What is more, model scores derived from ResNet of abnormal lung regions were obviously higher than those of healthy areas; Figure S2 presented the differentiation, indicating the discriminative capability of the model in radiological interpretation. Confusion matrices in this task were provided in Figure S3.

### 2.4 Model performance in DR‐TB diagnosis

**FIGURE 4:**
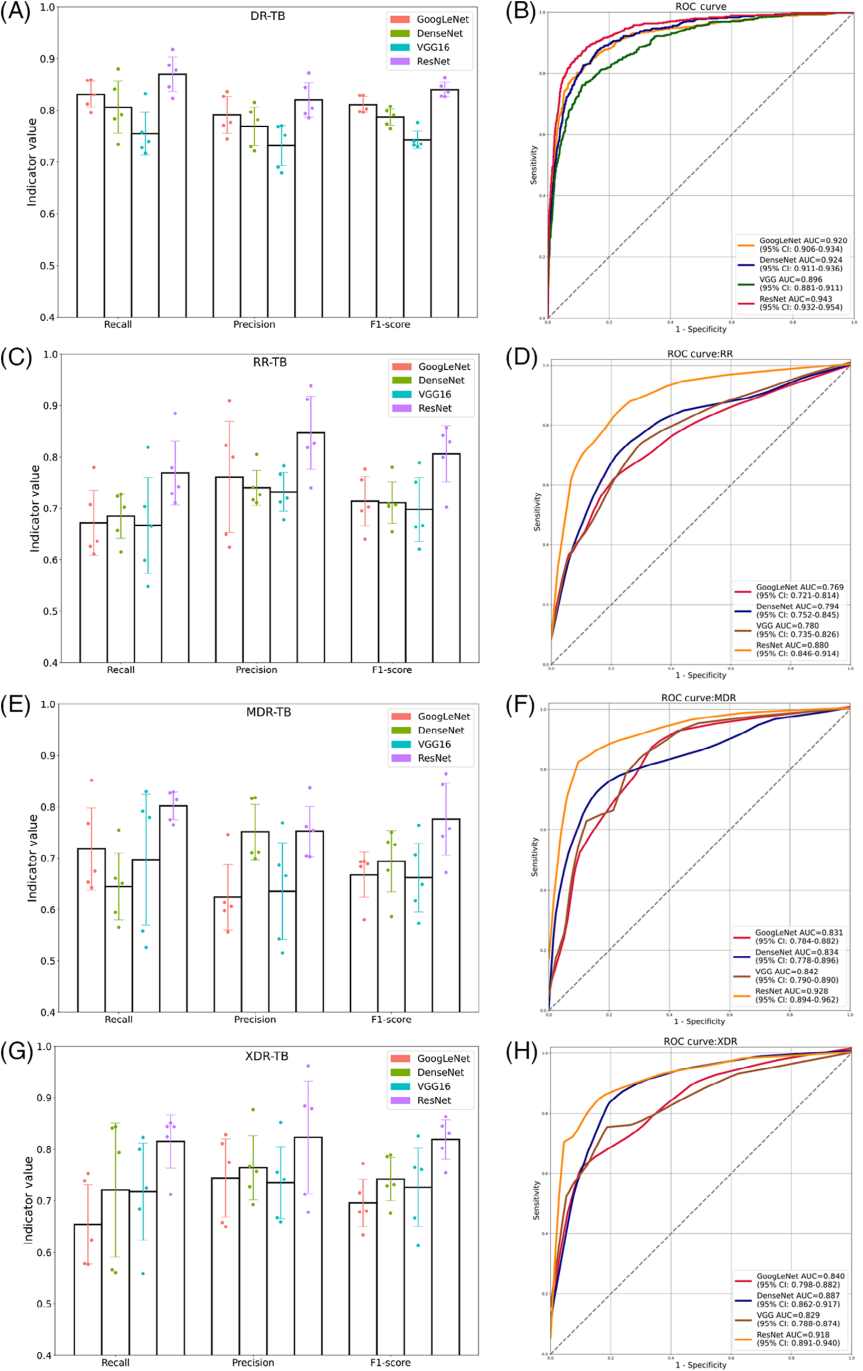
Recall, precision, F1‐score, and receiver operating characteristic (ROC) curves of four models in drug‐resistant tuberculosis (DR‐TB) diagnosis (A and B) and subtype triage of rifampicin‐resistant tuberculosis (RR‐TB) (C and D), multidrug‐resistant tuberculosis (MDR‐TB) (E and F), and extensively drug‐resistant tuberculosis (XDR‐TB) (G and H) in the test set.

Then, we conducted a binary task to explore the potential of the four CNNs to support an accurate diagnosis of DR‐TB. We calculated and compared intuitive measures, including recall, precision, and F1‐score, of the four models (Figure 4A). In the test set, with a recall of 0.870 (95% CI: 0.834, 0.899), a precision of 0.820 (95% CI: 0.787, 0.853), and the highest F1‐score of 0.840 (95% CI: 0.831, 0.858), ResNet showed the greatest diagnostic potential, surpassing the other three models, along with the highest AUC of 0.943 (95% CI: 0.932, 0.954). Moreover, it was satisfying that the other three models all performed well with AUCs of around 0.9 in this task as that GoogLeNet, DenseNet, and VGG16 achieved respective AUC at 0.920 (95% CI: 0.906, 0.934), 0.924 (95% CI: 0.911, 0.936), and 0.896 (95% CI: 0.881, 0.911) (Figure 4B).

In addition, ResNet achieved an accuracy of 0.915 (95% CI: 0.904, 0.925) and a specificity of 0.938 (95% CI: 0.928, 0.949), appropriately balancing false negatives and false positives combined with a recall exceeding 0.85. Although the other three networks attained specificity over 0.9, corresponding recall values were less than ResNet, suggesting worse sensitivity (Table 3). The confusion matrices were displayed in Figure S4.

**TABLE 3:** Performance of four models in drug‐resistant diagnosis and subtype classification in the test set.

Diagnosis	Model	Accuracy (95% CI)	Recall (95% CI)	Specificity (95% CI)	Precision (95% CI)	F1‐score (95% CI)	AUC (95% CI)
DR‐TB	GoogLeNet	0.901 (0.890, 0.913)	0.831 (0.796, 0.845)	0.928 (0.917, 0.939)	0.791 (0.757, 0.827)	0.811 (0.796, 0.826)	0.920 (0.906, 0.934)
	DenseNet	0.892 (0.880, 0.904)	0.806 (0.741, 0.841)	0.922 (0.910, 0.933)	0.769 (0.733, 0.806)	0.787 (0.772, 0.803)	0.924 (0.911, 0.936)
	VGG16	0.874 (0.862, 0.887)	0.755 (0.721, 0.802)	0.908 (0.896, 0.921)	0.732 (0.693, 0.770)	0.743 (0.726, 0.760)	0.896 (0.881, 0.911)
	ResNet	**0.915** (0.904, 0.925)	**0.870** (0.834, 0.899)	**0.938** (0.928, 0.949)	0.820 (0.787, 0.853)	0.840 (0.831, 0.858)	**0.943** (0.932, 0.954)
RR‐TB	GoogLeNet	0.674 (0.597, 0.691)	0.672 (0.584, 0.709)	0.751 (0.693, 0.807)	0.761 (0.615, 0.831)	0.714 (0.641, 0.736)	0.769 (0.721, 0.814)
	DenseNet	0.667 (0.610, 0.702)	0.685 (0.641, 0.727)	0.808 (0.747, 0.849)	0.740 (0.714, 0.781)	0.711 (0.661, 0.741)	0.794 (0.752, 0.845)
	VGG16	0.679 (0.621, 0.718)	0.667 (0.621, 0.807)	0.761 (0.734, 0.824)	0.732 (0.695, 0.771)	0.698 (0.603, 0.727)	0.780 (0.735, 0.826)
	ResNet	0.781 (0.741, 0.824)	0.769 (0.714, 0.838)	0.811 (0.764, 0.878)	0.847 (0.739, 0.881)	0.806 (0.744, 0.854)	**0.880** (0.846, 0.914)
MDR‐TB	GoogLeNet	0.710 (0.661, 0.756)	0.718 (0.680, 0.839)	0.781 (0.749, 0.814)	0.624 (0.559, 0.686)	0.668 (0.627, 0.715)	0.831 (0.784, 0.882)
	DenseNet	0.712 (0.643, 0.761)	0.645 (0.611, 0.741)	0.740 (0.721, 0.781)	0.751 (0.684, 0.791)	0.694 (0.625, 0.742)	0.834 (0.778, 0.896)
	VGG16	0.680 (0.633, 0.720)	0.697 (0.589, 0.846)	0.850 (0.824, 0.878)	0.636 (0.512, 0.699)	0.662 (0.598, 0.731)	0.842 (0.790, 0.890)
	ResNet	0.812 (0.770, 0.845)	0.802 (0.771, 0.824)	**0.866** (0.841, 0.889)	0.752 (0.712, 0.810)	0.776 (0.684, 0.824)	**0.928** (0.894, 0.962)
XDR‐TB	GoogLeNet	0.728 (0.691, 0.761)	0.654 (0.624, 0.778)	0.784 (0.729, 0.848)	0.744 (0.569, 0.821)	0.696 (0.627, 0.718)	0.840 (0.798, 0.882)
	DenseNet	0.772 (0.722, 0.818)	0.721 (0.620, 0.879)	0.791 (0.742, 0.845)	0.764 (0.697, 0.821)	0.742 (0.713, 0.797)	0.887 (0.862, 0.917)
	VGG16	0.721 (0.674, 0.769)	0.718 (0.671, 0.859)	0.838 (0.793, 0.881)	0.735 (0.651, 0.790)	0.726 (0.647, 0.798)	0.829 (0.788, 0.874)
	ResNet	0.827 (0.789, 0.868)	0.815 (0.811, 0.914)	**0.902** (0.863, 0.937)	0.823 (0.675, 0.892)	0.819 (0.763, 0.840)	**0.918** (0.891, 0.940)

The highest AUC value, along with corresponding accuracy, recall, and specificity when they were over 0.85 in DR‐TB diagnosis and subtype triage were bolded.

Abbreviations: AUC, area under the curve; CI, confidence interval; DR‐TB, drug‐resistant tuberculosis; MDR‐TB, multidrug‐resistant tuberculosis; RR‐TB, rifampicin‐resistant tuberculosis; XDR‐TB, extensively drug‐resistant tuberculosis.

### 2.5 Model performance in DR‐TB subtype triage

Notably, concerning the classification of the three major subtypes of DR‐TB to direct individualized anti‐TB treatment planning, the model based on ResNet also exerted highly instructive value (Table 3). In detail, ResNet reached recalls at 0.769 (95% CI: 0.714, 0.838), 0.802 (95% CI: 0.771, 0.824), and 0.815 (95% CI: 0.811, 0.914); precisions at 0.847 (95% CI: 0.739, 0.881), 0.752 (95% CI: 0.712, 0.810), and 0.823 (95% CI: 0.675, 0.892); F1‐scores at 0.806 (95% CI: 0.744, 0.854), 0.776 (95% CI: 0.684, 0.824), 0.819 (95% CI: 0.763, 0.840) for RR‐TB, MDR‐TB, and XDR‐TB categorizing, respectively. In the ROC comparison, ResNet still held the highest AUCs of 0.880 (95% CI: 0.846, 0.914), 0.928 (95% CI: 0.894, 0.962), and 0.918 (95% CI: 0.891, 0.940) for RR‐TB (Figures 4C and D), MDR‐TB (Figured 4E and F), and XDR‐TB classification (Figures 4G and H). Figure S5 provided confusion matrices of the four models in this task.

**FIGURE 5:**
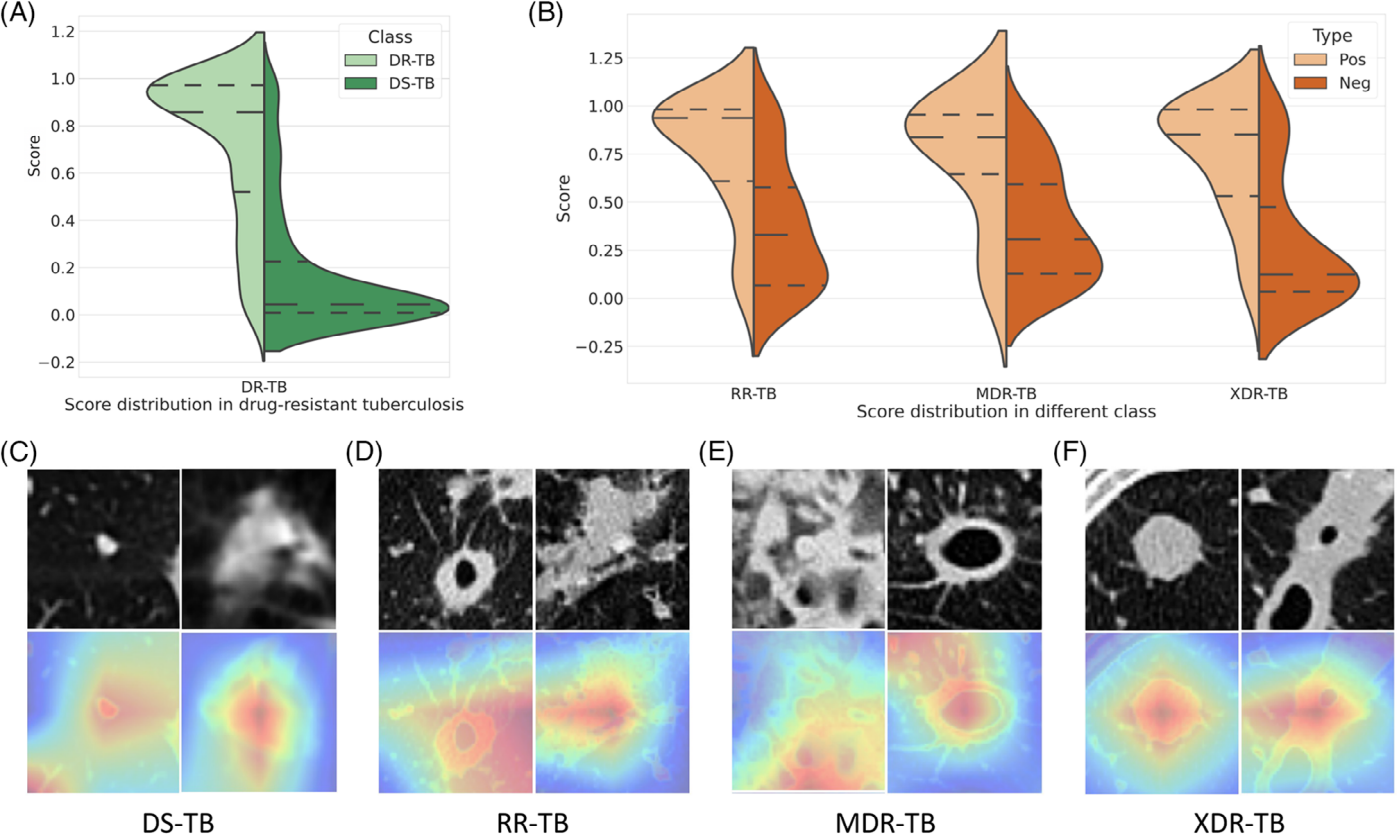
Model scores predicting drug‐resistant tuberculosis (DR‐TB) (A), rifampicin‐resistant tuberculosis (RR‐TB), multidrug‐resistant tuberculosis (MDR‐TB), and extensively drug‐resistant tuberculosis (XDR‐TB) (B) of DeepTB and representative class activation maps for visualization of lesions of drug‐sensitive tuberculosis (DS‐TB) (C), RR‐TB (D), MDR‐TB (E), and XDR‐TB (F). The images in each letter were derived from different patients. Neg, negative; Pos, positive.

Thus, after the above‐mentioned synthesized comparison, the ResNet‐based model was chosen as the optimal network and was named DeepTB. The model scores of the system in predicting patients with DR‐TB, RR‐TB, MDR‐TB, and XDR‐TB were significantly higher than negative ones, showing a decent recognition capacity (Figures 5A and B).

### 2.6 Model visualization via class activation maps

In realistic clinical settings, due to the opaque black‐box effect of CNNs, physicians may be suspicious of the model output.^29^ Thus, the algorithm generated class activation maps (CAMs) to precisely locate the anomalous foci in the lung region, translating the model output into a human‐understandable visual concept. Characteristics, such as nodule, patchy shadow, cavity, and consolidation, which may contribute to the final prediction, were strongly activated (Figures 5C–F). Thus, CAMs provided a robust and explainable result by visualizing of the rationale behind the generation of the model, through which the human experts could comprehend how the system achieved the final verdict.

## 3 DISCUSSION

In this investigation, actuated by the desire for precise identification of DR‐TB to assist in achieving the vision of eliminating TB worldwide, we developed a clinically applicable deep learning system, DeepTB, to efficiently analyze chest abnormality, DR‐TB suffering, and the specific kind of subtype by decoding CT volumes via the pretrained CNN backbone, showing remarkable performance improvement.

Therapeutic regimens of DR‐TB are different from DS‐TB and vary widely upon particular subtypes.^30^ Emerging evidence has revealed higher DR‐TB morbidity in previously treated patients, implying that optimal regimens in the beginning may arrest the disease progression to an expanded drug resistance spectrum.^31^, ^32^ The curative effect of certain drugs has been authenticated and regimens including specific ones, such as bedaquiline, linezolid, clofazimine, later‐generation fluoroquinolones, and carbapenems, may improve the therapeutic efficiency of DR‐TB, which could be a reference to clinical decisions.^33^ Therefore, precise diagnosis of DR‐TB is crucial for proper treatment initiation, with further objectives to ameliorate clinical outcomes and maintain public health welfare.^34^

Radiology, a comparatively more convenient and time‐saving strategy for disease assessment, demonstrates enormous potential in DR‐TB evaluation.^35^ A logistic regression analysis has been carried out based on the differential representations on chest CT scans between DR‐TB and DS‐TB. Various characteristics, including wider lung involvement range, emphysema, bronchial dissemination, multiple cavities, exudative lesions, and so on, may participate in DR‐TB prediction.^13^ Additionally, in specific DR‐TB subtypes, features of lesion range and cavities manifest discriminatively as well. In RR‐TB, small cavities with diameters less than 1.5 cm are commonly seen, and the patients with MDR‐TB often present more than three cavities. XDR‐TB, the most serious one, can demonstrate lesions that spread to more than three lung fields even TB‐destroyed lung.^12^ Thus, CT imaging deserves extended exploration in diagnostic usage.

Deep learning, a major branch of AI,^36^ is experiencing an era of explosive growth, constituting a breakthrough in medical image classification tasks by mining the association between raw input visual data and desired output, generating decisions ranging from macroscopic disease diagnosis and outcome prediction to microscopic gene mutation status.^37^, ^38^, ^39^, ^40^, ^41^, ^42^, ^43^, ^44^, ^45^, ^46^, ^47^, ^48^, ^49^ Along with the vigorous evolution of AI techniques, medical image‐based computational approaches have been proposed for TB, showing comparable performance to radiologists in disease assessment.^15^, ^16^, ^17^, ^18^, ^19^, ^20^ And efficiency of software for automated TB diagnosis has been validated, offering novel insights for TB management.^50^, ^51^ Moreover, we constructed a high‐profile deep learning pipeline to classify several pulmonary diseases, including TB, achieving a diagnostic performance approximate to senior radiologists.^46^

In terms of DR‐TB, the existing situation is not so desirable. AI systems that achieved preferable outcomes mostly were trained by gene sequences of *Mycobacterium tuberculosis* isolates,^52^, ^53^, ^54^, ^55^ not applicable to resource‐limited settings on account of the costly sequencing facility requirement. As for medical image‐based studies, alarming issues, including limited datasets, moderate performance, and rough subtype classification, surround the implementation of these AI models into real‐world medical utility and precise medicine.^22^, ^23^, ^48^, ^56^ In addition, most of these models were developed by CXRs, rather than CT volumes, which demonstrate more differential and subtle features of intricate tissue structures and are of more significant value in diagnosis.^57^ In a study that utilized CT images to identify DR‐TB, the dataset contained over 200 patients and the DR‐TB cohort solely consisted of MDR‐TB, without the subtype mentioned.^22^

To a certain extent, the strategy applied in our study surmounts the abovementioned conundrums. We have made progress for DR‐TB diagnosis and subtype triage before.^58^ And in this study, based on a dataset of CT volumes from more than 1000 patients, we further involved four deep learning architectures and compared their performance via various parameters. For model performance improvement, three strategies were adopted. First, we conducted pretraining via transfer learning,^59^ leveraging the knowledge learned from the large‐scale dataset, Kinetics, and obtained an accelerated convergence speed. Moreover, as conventional large dataset‐based supervised learning mostly depends on high‐quality labels hand‐assigned by researchers, demanding a considerable amount of well‐trained experts and work,^60^ we utilized the semi‐supervised learning approach,^61^ through which the model performance could be enhanced based on unlabeled information. In contrast to the traditional pseudo‐label generation methods, we ensured reliability by calculating the informative of samples and partitioning the data. Combined with K‐nearest neighbor‐equipped CNNs, confirmation bias issues could be alleviated, outperforming threshold‐based conventional pseudo‐labeling. Finally, we integrated auxiliary information through the multitask learning strategy to assist primary task training.^62^ Through optimization of multiple loss functions, the network could extract a shared feature set relevant to both tasks to improve the performance of predominant task.

ResNet, of which the core mechanism is the residual connection, assisting in addressing the issue of gradient vanishing and enabling deeper network training without a substantial increase in parameters, was selected as the optimal backbone for DeepTB construction. And this advantage became more apparent in the 3D data we were dealing with. Additionally, global average pooling of ResNet could reduce overfitting and performed well in transfer learning and fine‐tuning in our tasks, meanwhile exhibiting robust performance in handling noisy or incomplete data.

Therefore, DeepTB presents great potential to be used as the frontline tool for streamlining DR‐TB care, especially after being integrated into clinical workflow. To achieve this assumption, embedding the model into a software or online webserver, which is smoothly linked to the procedures of CT examination and image generation, is a practical strategy. After the CT volumes were input, the algorithm was capable of precisely localizing the chest lesions and recognizing corresponding anomalies. With the assistance of DeepTB, radiologists can utilize the model output as a reference for image interpretation with higher efficiency in a routine process. If there is any disagreement between human specialists and the machine, the radiologists may decide whether or not to change the findings after reassessment, through which the reading mistakes could be reduced. Then, the system automatically generated an accurate DR‐TB prediction and specific subtypes, facilitating individual regimen planning upon different drug resistance profiles. In addition, we utilized CAMs to visualize the inner working of DeepTB via the depiction of abnormal chest areas, explaining the mystique of how the results were derived from an image‐based deep learning system. And the Circos which demonstrated the correlation between chest abnormalities and drug resistance state suggested the diagnostic significance of those radiological findings, as well as the underlying effect on lung damage of different *Mycobacterium tuberculosis* resistant mutants.

There are several limitations in our study. First, the training data were extracted from a single hospital, and included participants containing Asia people only, leading to a potential bias and restricting the generalization of the model in unrelated population. Enrolling individuals from multicenter organizations with various geography, demographics, and disease spectrum may help to ensure the accuracy of the results and model popularization. Moreover, the dataset remains to be expanded for model optimization in the era of big data. In the meantime, to improve the utility of DeepTB, prospective validation and clinical trials are essential to solve the shortage of the retrospective study. Then, as the DR‐TB proportion in this study was higher than in reality, the model performance needs to be evaluated in a TB cohort with a lower DR‐TB prevalence approximate to the real world. In addition, we utilized CT volumes for the model development only, while analyzing multidimensional medical data is required in clinical reality. Besides radiology, integrating medical records, laboratory tests, pathology, and so on can provide a more precise decision. Multimodal deep learning strategy becomes an emerging method in healthcare to realize this vision. Our team has built a unified model for evaluating respiratory diseases by embedding visual and textual information, outperforming the image‐only algorithm and shedding light on the future strategy applied in DR‐TB.^42^ Finally, it should be stressed that radiology and AI just serve as auxiliary diagnostic tools of DR‐TB, while molecular drug resistance testing still is the gold standard for definite diagnosis.

## 4 CONCLUSIONS

To sum up, DeepTB demonstrates tremendous potential in DR‐TB management on the basis of deep learning strategy. The algorithm precisely identifies the typical TB chest anomalies on CT scans, showing a latent capacity to reduce diagnostic workflow time costs and the need for diagnostic expertise in radiological explanation, the critical process of disease evaluation. Subsequently, the output of DR‐TB diagnosis and subtypes can assist clinicians in precise medication initiation and avoid improper drug usage. Showing prominent accuracy, DeepTB is helpful in improving the treatment enrollment and the life quality of patients with DR‐TB, especially in settings with limited resources and a high TB incidence. Going forward, automated systems for treatment assessment and clinical outcome prediction of DR‐TB are warranted. Additionally, it is worthwhile to reflect on how to convert AI prototypes into practical clinic systems.

## 5 MATERIALS AND METHODS

### 5.1 Study participants

We randomly enrolled 1751 patients with pulmonary TB who visited West China Hospital of Sichuan University between January 2008 and November 2023 retrospectively. This study was approved by the institutional review boards of West China Hospital of Sichuan University, with a waiver of the requirement for informed consent from each patient.

The inclusion criteria were as follows: (1) patients who were confirmed with TB through pathogenic detection (microscopy or culture of *Mycobacterium tuberculosis*) or clinical confirmation through a synthesized analysis of radiology, symptoms, and laboratory tests; (2) the results of DST, GeneXpert MTB/RIF, or gene testing for drug resistance detection for DR‐TB and above results or follow up information for DS‐TB were available; (3) patients who were 18−90 years old. The exclusion criteria were: (1) CT images within 3 months of diagnosis were unavailable; (2) incomplete CT images; (3) accompanying other severe lung diseases that were confusing on CT volumes, such as severe pneumonia, pulmonary abscess, lung cancer, pulmonary fibrosis, sarcoidosis, and so on. Ultimately, 1176 patients were eligible for this study (Table 1 and Figure S1).

### 5.2 Image preprocessing

The selected CT images were input into the annotation system for processing. We conducted data transfer before manual annotation. In this procedure, we applied filtering to all CT data for each patient based on various fields, including CT series number, window width, window center, and acquisition date, which were obtained from the digital imaging and communications in medicine (DICOM) data. Specifically, we excluded the image data that did not match the CT sequence numbers and diagnosis time interval. And then we identified lung window CT images through window width and window center criteria. Next, we imported the filtered data into our database.

There were various irrelevant regions while the original CT volumes were utilized as the raw input, posing a significant challenge to the model training. To allow the model to focus more on TB‐related areas, we cropped out the lesions and selected the largest plane for each lesion as the central plane. As CT volumes were presented in a 3D form, we calculated the depth of each lesion as half the sum of its length and width. This calculation was performed to crop the lesion into a 3D cube shape and then we employed a resampling algorithm to adjust the pixel spacing to 1 × 1 × 1. In detail, we retrieved the pixel spacing information from the DICOM data. Then, we obtained the scaling factor to adjust the original pixel spacing to the target spacing by dividing the target spacing by the original spacing. Subsequently, we employed the trilinear interpolation algorithm to interpolate and calculate new pixel values in the original image. For each point in the target image to be resampled, the position of that point in the new image was calculated based on the scaling factor. For the target position to be interpolated, the weights of its nearest eight pixels in the original image were computed. We assigned the reciprocal of the distance between the interpolation point and the original image pixel as the weight for that pixel, with closer pixels having higher weights. Using the calculated weights, we performed interpolation for the target position. This process would be repeated for each position in the target image until the entire image was interpolated, thus achieving the adjustment of pixel spacing.

Then, we resized the cropped lesions with a consistent size in a width/height of 128 and a depth of 64 and input them into the algorithm for model training. These resized lesions were referred to as lesion‐based patches and were used to train the networks for chest abnormality identification and DR‐TB diagnosis. To address the imbalance in sample categories, all DR‐TB patches were employed while DS‐TB patches were randomly selected to ensure data balance between the two categories.

As the number of TB‐associated lesions varied among the study participants, we recorded the number of all lesions in each case. Then we calculated the partition length which was the value of the predefined depth divided by the amount of the lesions. We centered on half the depth of the lesion input and extracted slices extending half of the partition length both upwards and downwards along the depth axis to obtain the partitioned lesions. All partitioned lesions of each case were then stacked to obtain the predefined input depth size. These patches were utilized in the DR‐TB subtype classification. Data augmentation strategies, such as random rotation and flipping, were utilized to reduce the risk of overfitting.^63^

### 5.3 Model development

We proposed four patch‐based deep learning models to distinguish DR‐TB from DS‐TB automatically. The overall workflow of the model construction was depicted in Figure 1.

In this study, we employed a semi‐supervised learning approach for pseudo‐label generation, utilizing the feature information of unlabeled data to optimize the model performance. Simultaneously, considering the issue of data imbalance in our tasks, we adopted a sample informativeness‐based approach.^64^ By treating highly informative labeled samples as anchor samples, we calculate the informativeness of unlabeled samples by measuring their similarity to these anchor samples. We emphasized unlabeled samples with higher informativeness, which were positioned as far as possible from the distribution of labeled samples, making them likely to belong to the minority. This strategy addressed the issue of class imbalance during training. Additionally, the method combining model predictions with K‐nearest neighbor predictions guided by sample informativeness effectively alleviated confirmation bias issues.^65^

To extract spatial information from CT images, we constructed 3D patch‐based automated systems based on four CNNs,^66^ including GoogLeNet,^67^ DenseNet,^68^ VGG16,^69^ and ResNet,^70^ to identify the different characteristics of chest lesions from patients with DS‐TB and DR‐TB. All four models were pretrained on a large‐scale image dataset Kinetic.^70^, ^71^ GoogLeNet replaced traditional convolutions with Inception modules and DenseNet utilized dense connections within the network to enhance model performance. VGG16 and ResNet respectively employed depth‐wise small convolutional kernels and residual connections to improve efficiency. The penalty cross‐entropy (PCE) loss [$l(y_{n},P_{n})]$ was used to distinguish false negatives from false positives by penalizing each error differently,^72^ as shown in the following equation:

$$l\;\left(y_{n},P_{n}\right)=\;-\delta_{n}\left[y_{n}\log\left(P_{n}\right)+\left(1-y_{n}\right)\log\left(1-P_{n}\right)\right]$$

In this equation, $\;y_{n}\;\mathrm{and}\;P_{n}$ referred to the true label and the predicted label of lesions, respectively. The penalty factor $\delta_{n}$ was calculated as follows:

$$\;\delta_{n}=\;\left\{\begin{array}{cc} C, & y_{n}-P_{n}>0.5\\ 1, & otherwise \end{array}\right.$$

where *C* represented the given penalty value which was set to 2 in our experiment.

Notably, we adapted chest abnormalities as auxiliary labels in DR‐TB diagnosis and subtype classification training via multitask learning. Ten neurons were used to output the morphological features of pulmonary lesions and one neuron was employed to generate the probability of DR‐TB. The multitask loss function was the weighted combination of the PCE loss and the binary cross‐entropy (BCE) loss with sigmoid. The PCE loss was used for the DR‐TB identification and triage, and the BCE loss with sigmoid was used for identifying morphological characteristics. The loss function of DR‐TB diagnosis and subtype classification was $L_{\mathrm{Dr}}$, and the loss function of chest abnormality classification was $L_{\mathrm{Morph}}$. The multitask loss function $\left(L_{\mathrm{multi}}\right)$ was defined as follows:

$$L_{\mathrm{multi}}=\alpha L_{\mathrm{Dr}}+\;\beta L_{\mathrm{Morph}}$$

where α and β denoted the weight factor of the classification tasks.

In subtasks, including chest abnormality detection, DR‐TB diagnosis, and subtype triage, each experiment was conducted five times, and the results were calculated by averaging the results of five experiments. All the experiments were conducted on an Ubuntu 18.04 server with one Tesla V100 (32G) GPU using CUDA 10.1. The model was coded and built using Python 3.6 and PyTorch 1.0.0 and the model was trained with 120 epochs with the stochastic gradient descent (SGD) algorithm employed as the optimizer. The learning rate (LR) was set to 0.006 and divided by 10 after every 50 epochs.

### 5.4 Statistical analysis

AUC, accuracy, specificity, recall (sensitivity), precision, F1‐score, and confusion matrix were utilized to evaluate the model performance by Python 3.6 open resource package. AUC can synthetically assess the model's ability in object prediction, indicating better model performance with a value closer to 1. Specificity refers to the ratio of correctly predicted negative samples.^73^ Recall (sensitivity) is the proportion of correctly predicted positive samples and precision presents the ratio of samples predicted as positive by the model that are true positive, while the F1‐score is the harmonic mean of precision and recall.^74^ These metrics provided a comprehensive evaluation of the proposed models. The 95% CIs were calculated by the Delong method.

## AUTHOR CONTRIBUTIONS

Chengdi Wang and Weimin Li conceived the concept for the study. Shufan Liang and Jun Shao selected the participants and annotated the imaging data. Xiuyuan Xu, Zhe Yang, Qiuyu Du, Lingyu Zhou, and Jixiang Guo contributed to the algorithm development. Shufan Liang, Xiuyuan Xu, and Zhe Yang drafted the original version of the manuscript and designed the figures and tables. Chengdi Wang and Binwu Ying revised the final submission. All authors have read and approved the final manuscript.

## CONFLICT OF INTEREST STATEMENT

The authors declare that the research was conducted in the absence of any commercial or financial relationships that could be construed as a potential conflict of interest.

## ETHICS STATEMENT

Institutional Review Board approval was obtained with the approval number of 2023 (2286).

## Supporting information

Supporting Information

## Data Availability

All the data are available from corresponding authors upon reasonable request.
