# Supplementary material for: Deep learning for precise diagnosis and subtype triage of drug‐resistant tuberculosis on chest computed tomography
Source: MedComm (2020). 2024 Mar 10;5(3):e487. doi: 10.1002/mco2.487 (PMC10925488; doi:10.1002/mco2.487)
Supplement: Supplementary file 1 — Supporting Information [file MCO2-5-e487-s001.docx]

Title Page

**Deep learning for precise diagnosis and subtype triage of drug-resistant tuberculosis on chest computed tomography**

Shufan Liang^1#^, Xiuyuan Xu^2#^, Zhe yang^2^, Qiuyu Du^2^, Lingyu Zhou^2^, Jun Shao^1^, Jixiang Guo^2^, Binwu Ying^3^, Weimin Li^1*^, Chengdi Wang^1*^

^1^Department of Pulmonary and Critical Care Medicine, State Key Laboratory of Respiratory Health and Multimorbidity, Targeted Tracer Research and Development Laboratory, Med-X Center for Manufacturing, Frontiers Science Center for Disease-related Molecular Network, West China Hospital, West China School of Medicine, Sichuan University, Chengdu, China

^2^Machine Intelligence Laboratory, College of Computer Science, Sichuan University, Chengdu, China

^3^Department of Laboratory Medicine, West China Hospital, Sichuan University, Chengdu, China

*Correspondence

Chengdi Wang, Department of Pulmonary and Critical Care Medicine, State Key Laboratory of Respiratory Health and Multimorbidity, Targeted Tracer Research and Development Laboratory, Med-X Center for Manufacturing, Frontiers Science Center for Disease-related Molecular Network, West China Hospital, West China School of Medicine, Sichuan University, Chengdu 610041, China.

Email: [chengdi_wang@scu.edu.cn](mailto:chengdi_wang@scu.edu.cn)

Weimin Li, Department of Pulmonary and Critical Care Medicine, State Key Laboratory of Respiratory Health and Multimorbidity, Targeted Tracer Research and Development Laboratory, Med-X Center for Manufacturing, Frontiers Science Center for Disease-related Molecular Network, West China Hospital, West China School of Medicine, Sichuan University, Chengdu 610041, China.

E-mail address: [weimi003@scu.edu.cn](mailto:weimi003@scu.edu.cn)

**Supplementary figures**


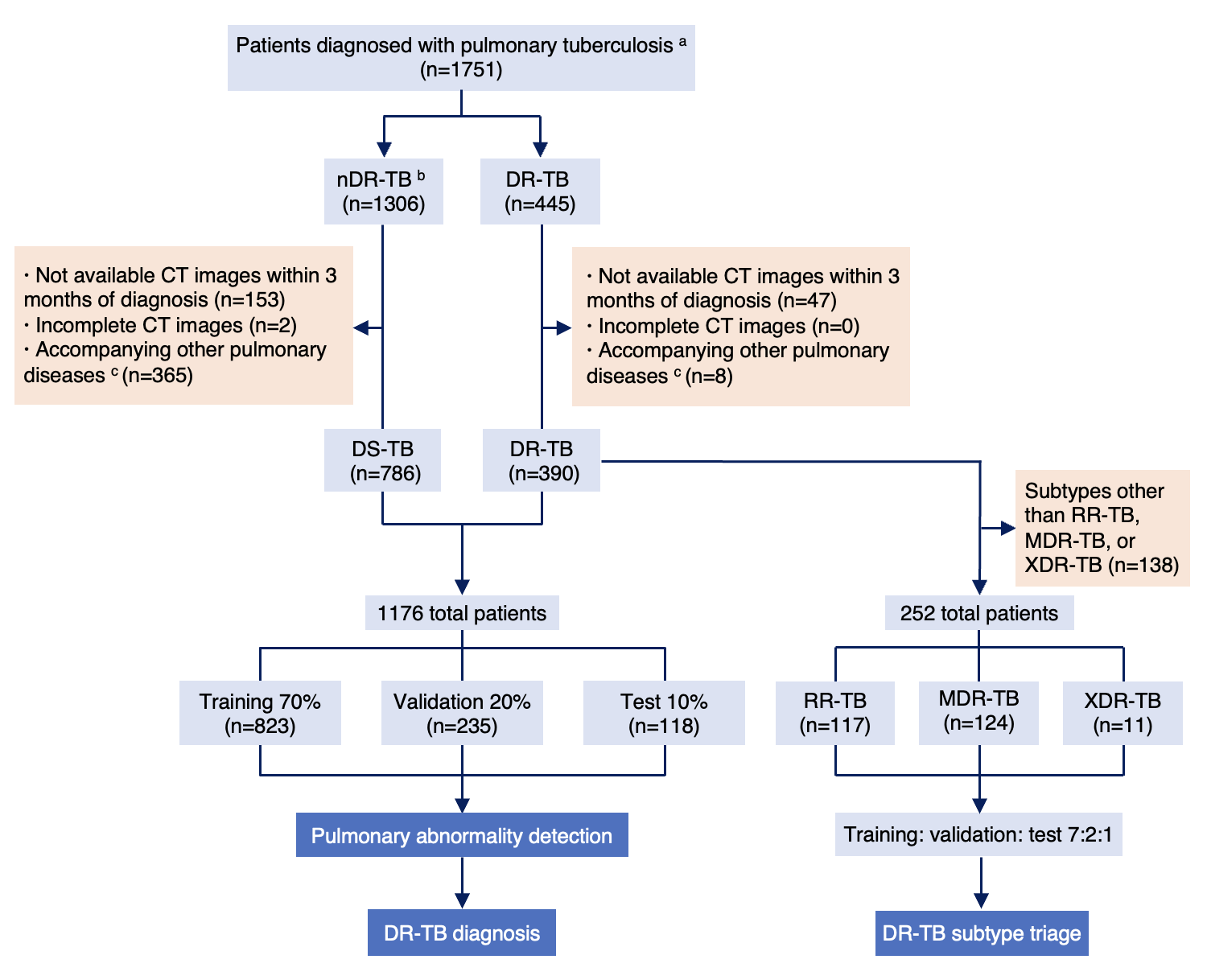


**Figure S1.** Diagram of participant selection. CT, computed tomography; DR-TB, drug-resistant tuberculosis; DS-TB, drug-sensitive tuberculosis; MDR-TB, multidrug-resistant tuberculosis; RR-TB, rifampicin-resistant tuberculosis; XDR-TB, extensively drug-resistant tuberculosis.

^a^ Patients were diagnosed by (1) microscopy or culture of *Mycobacterium tuberculosis* or (2) clinical confirmation through a synthesized analysis of radiology, symptoms, and laboratory tests); ^b^ nDR-TB refers to patients with no evidence of DR-TB and improved or stable condition during follow-up; ^c^ Severe pneumonia, pulmonary abscess, lung cancer, pulmonary fibrosis, and sarcoidosis, etc. of main diagnosis.


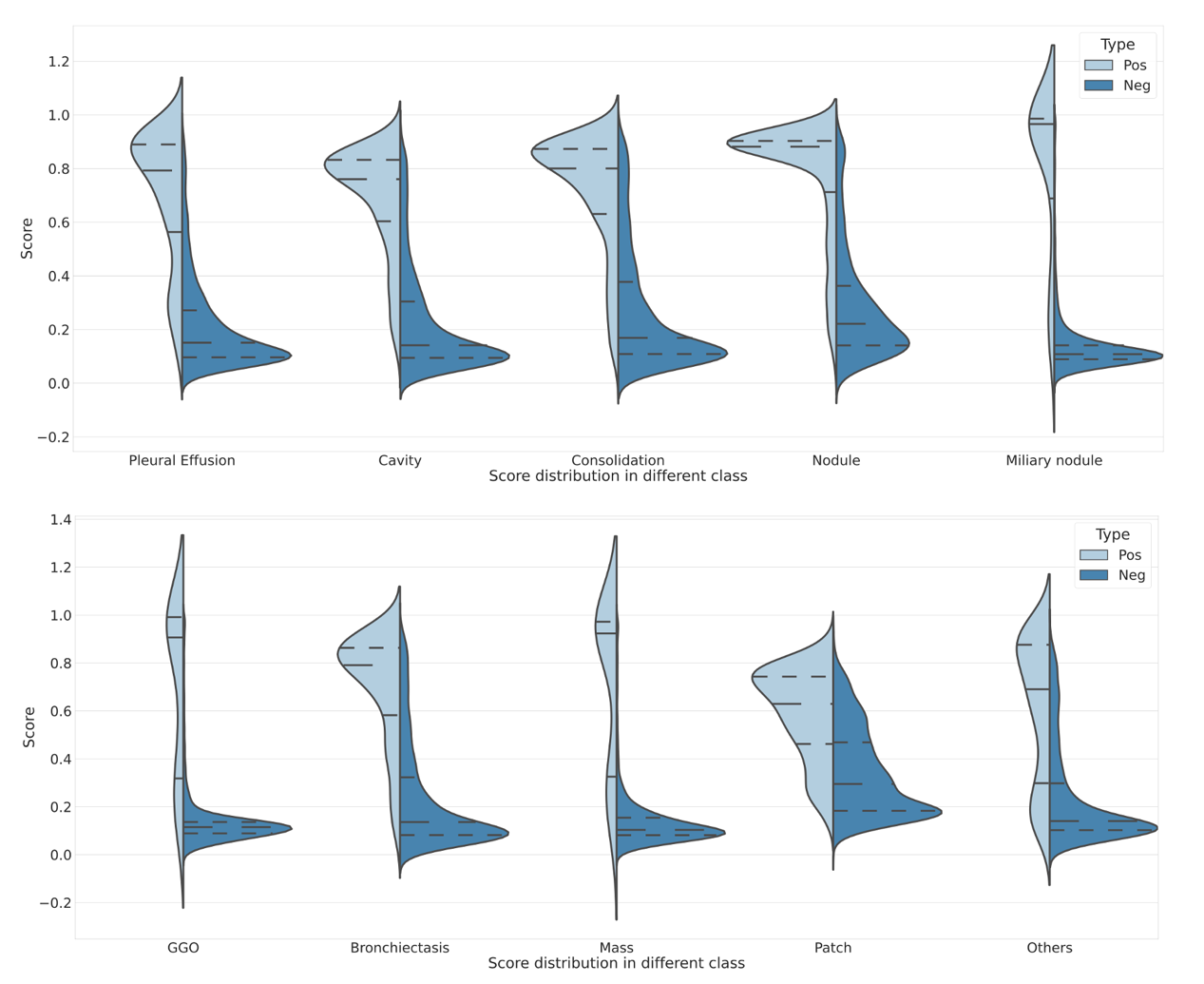


**Figure S2.** Model scores in chest abnormality detection of DeepTB. GGO, ground-glass opacity; Neg, negative; Pos, positive


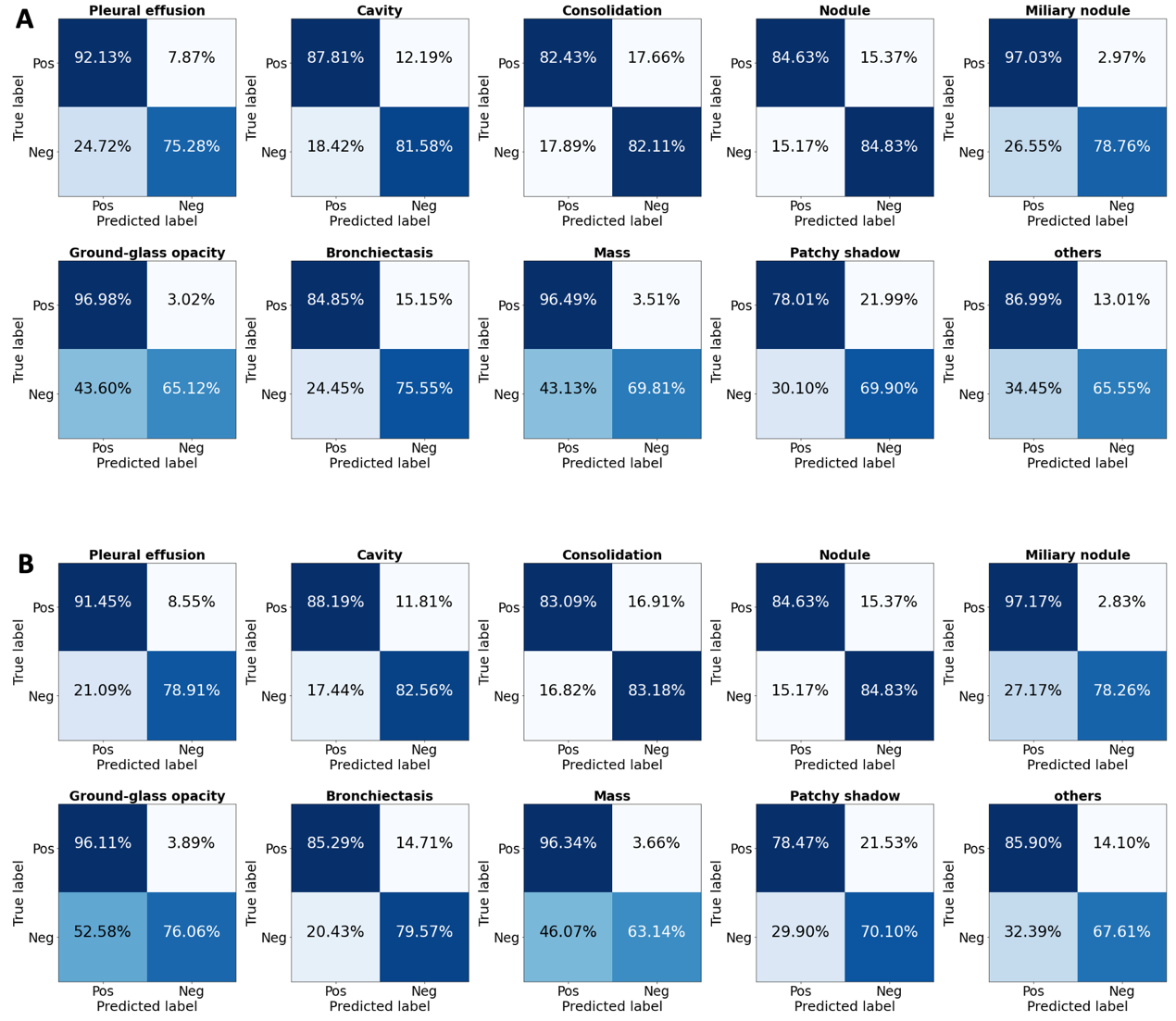


**Figure S3.** Confusion matrices of the DeepTB to identify chest abnormalities in the (A) validation and (B) test set. Neg, negative; Pos, positive.


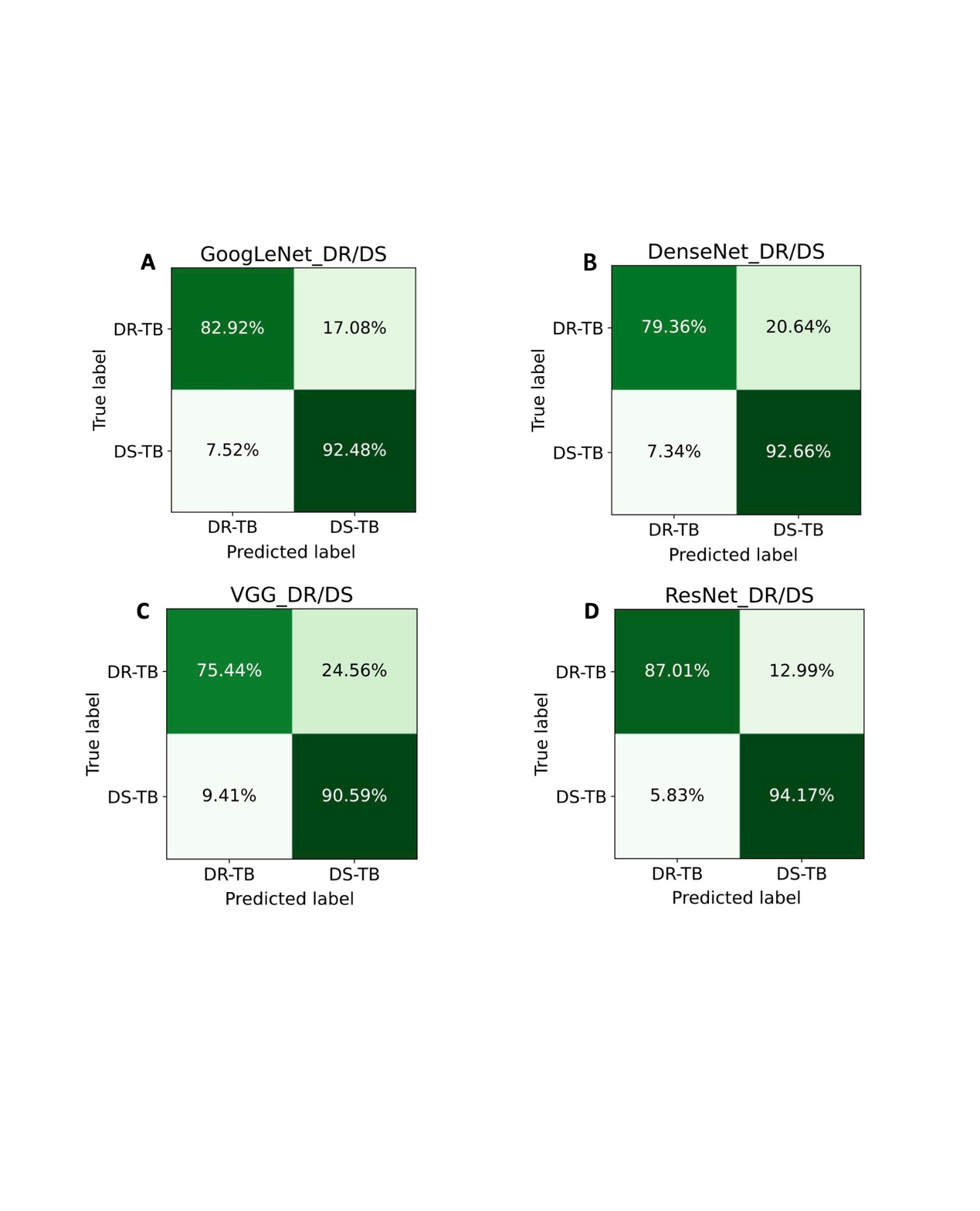


**Figure S4.** Confusion matrices of the (A) GoogleNet, (B) DenseNet, (C) VGG16, and (D) ResNet to discriminate drug-resistant tuberculosis (DR-TB) from drug-sensitive tuberculosis (DS-TB).


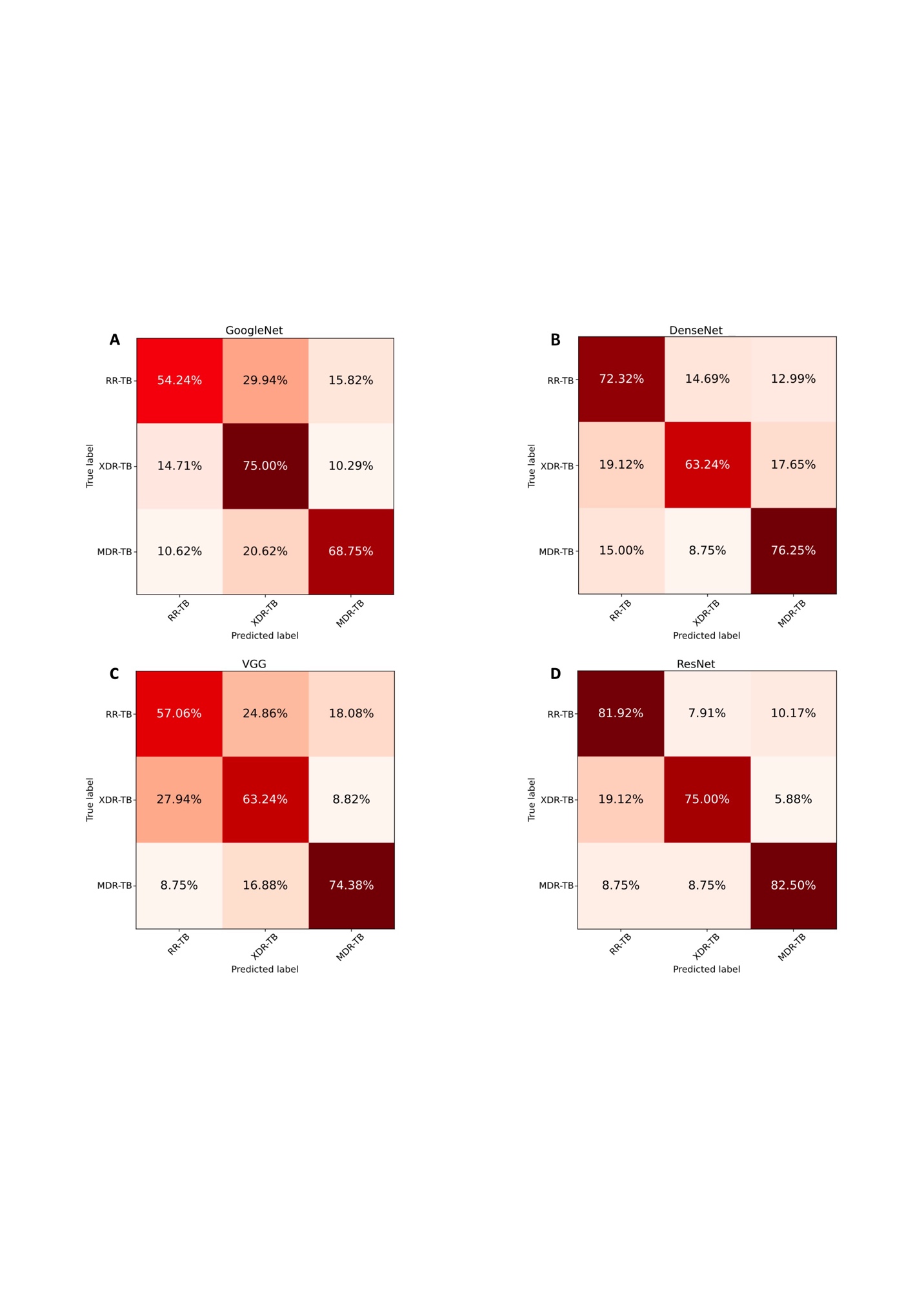


**Figure S5.** Confusion matrices of the (A) GoogleNet, (B) DenseNet, (C) VGG16, and (D) ResNet to classify the three major subtypes of drug-resistant tuberculosis (DR-TB). MDR-TB, multidrug-resistant tuberculosis; RR-TB, rifampicin-resistant tuberculosis; XDR-TB, extensively drug-resistant tuberculosis.
